# Systemic amyloidosis: an aggressive evolution in a patient with relapsing polychondritis and monoclonal gammopathy of undetermined significance (MGUS) undergoing peritoneal dialysis

**DOI:** 10.4322/acr.2024.518

**Published:** 2024-09-27

**Authors:** Thiago Cavalcanti Matos, William George Giusti Fischer, Rosa Maria Rodrigues Pereira, Andre Silva Franco

**Affiliations:** 1 Universidade de São Paulo (USP), Faculdade de Medicina, Hospital das Clínicas, Serviço de Reumatologia, São Paulo, SP, Brasil; 2 Universidade de São Paulo (USP), Faculdade de Medicina de Ribeirão Preto, Hospital das Clínicas, Serviço de Hematologia, Ribeirão Preto, SP, Brasil

**Keywords:** Amyloidosis, Paraproteinemias, Polyneuropathies, Autonomic Nervous System Diseases

## Abstract

Herein, we report the case of primary amyloidosis with multi-organ involvement in a female patient in her 50s. The patient had a history of relapsing polychondritis, chronic kidney disease, and monoclonal gammopathy of undetermined significance (MGUS). The clinical manifestations included neuropathic pain, sensorimotor polyneuropathy, intrahepatic cholestatic liver injury, gastrointestinal symptoms, dysautonomia, and myocardial thickening. Initial histologic evaluations of the abdominal fat pad aspirate and bone marrow biopsy were negative for amyloid deposition. However, due to a high index of suspicion, a second bone marrow biopsy was performed, confirming the presence of the amyloid protein. Given the patient's complex medical history, other types of amyloidosis, such as AA amyloidosis, AL amyloidosis, and ß2-microglobulin amyloidosis, should also be considered as differential diagnoses. The type of amyloid protein was subsequently identified through laser microdissection of amyloid fibrils followed by liquid chromatography–tandem mass spectrometry as AL-lambda (amyloid light-chain) amyloidosis. The patient presented unfavorable evolution, with progressive dysautonomia, being admitted to the ICU, culminating in refractory circulatory shock, and undergoing an empirical broad-spectrum antibiotic therapy. After a few days, she presented pulseless ventricular tachycardia, culminating in her death, before undergoing specific treatment. This article highlights the crucial role of precise identification in guiding appropriate therapeutic strategies for this complex, yet potentially severe, diseases.

## INTRODUCTION

Amyloidosis is a heterogeneous group of more than 30 diseases characterized by organized extracellular deposition of misfolded proteins in target organs, named firstly in 1854 by Rudolph Virchow.^1,2^ These proteins aggregates in the form of insoluble and resistant to proteolysis fibrils that result in tissue infiltration and swelling leading to progressive loss of function of the affected organ.^2^

Despite being underdiagnosed and reports consisting mostly of retrospective case series,^1,3^ some epidemiological population-based studies have been conducted. An incidence of AL amyloidosis estimated in 1.2 per 100.000 person-years was reported between 1950-2015 in Olmsted County, MN, USA, similar to a France study in Limousin region of 1,25 per 100.00 person-year between 2012-2016.^4^ The estimated global incidence of amyloidosis is five cases per million person-years in England^5^ and eight patients per million person-years in Sweden.^6^

These entities vary in classification and manifestations according to the type of protein forming the amyloid deposit. At least 36 different proteins are associated with amyloidosis, as described to date. Amyloid light chain (AL) amyloidosis, the most prevalent, is caused by excessive light chain production by abnormal proliferation of monoclonal B cells (typically plasmacytes).^2,7^ ATTR amyloidosis, the second most common, is caused either by an autosomal dominantly inherited point mutation of the precursor protein transthyretin (hereditary amyloidosis, ATTRv) or by a nonfamilial acquired form of normal TTR deposition (wild type amyloidosis, ATTRwt).^2^ Transthyretin is an acronym for the transport protein of thyroid hormone and retinol-binding protein. Secondary amyloid A amyloidosis (AA), third in prevalence, is related to chronic inflammation with deposition of amyloid A protein - primarily associated with rheumatoid arthritis, juvenile idiopathic arthritis, familial Mediterranean fever, and ankylosing spondylitis. Other familial amyloidosis include a1-apolipoprotein, gelsolin, among others.^7,8^ ß2-microglobulin-related amyloidosis, a rare type, is caused by the excessive deposition of a constitutional protein part of major histocompatibility complex (MHC) class I molecules with near total renal clearance but not fully dialysable on dialysis patients, especially the ones undergoing low-efficiency dialysis.^9,10^ The prognosis depends on staging and subtype, with a median survival of 6 to 12 months for AL amyloidosis, 3 to 4 years for AA amyloidosis, and almost 10 years for ATTR.^2,11^

**Figure 1:**
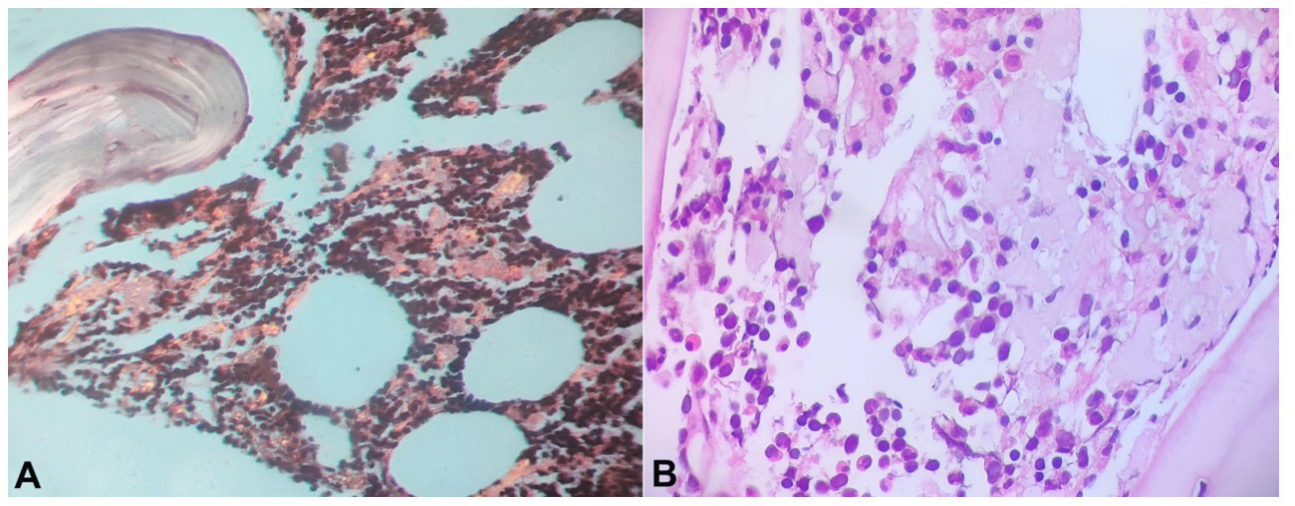
Timeline.

Despite pathogenesis similarities regarding clinical manifestations of protein deposition, such as proteinuria, organomegaly (liver, spleen, or tongue), right-sided cardiac failure and/or biventricular pseudo-hypertrophy of cardiac walls, orthostatic hypotension, peripheral axonal polyneuropathy, autonomic neuropathy, and malabsorption, and definite diagnosis consisting in detecting Congo red-positive extracellular deposits showing yellow birefringence under polarized light microscopy (Figure 1), therapeutic approaches vary greatly.^2^ It is also noteworthy that cardiac amyloidosis is often associated with low voltage QRS complexes on electrocardiogram, a finding unexpected in cases of true hypertrophy. Therefore, precisely identifying amyloidosis is crucial for guiding appropriate treatment. We illustrate a case that exemplifies the diagnostic complexity of this condition, where multiple possibilities of amyloidosis made it challenging to choose the correct treatment.

## CASE REPORT

A female patient in her 50s was admitted to our clinical ward to investigate 4 months of lower limbs neuropathic pain, 1 month of intrahepatic cholestatic pattern of liver injury (alanine transaminase 47 UI/L [reference value <39] / aspartate transaminase 125 UI/L [reference value <31] / gamma-glutamyl transferase 1034 UI/L [reference value 5-36] / alkaline phosphatase 719 UI/L [reference value 35-104]) and 10 days of alternating periods of constipation and diarrhea. On admission, she also presented signs of dysautonomia with significant postural hypotension and tachycardia at rest (sinus rhythmic heart rate of 114 beats/minute).

**Figure 2:**
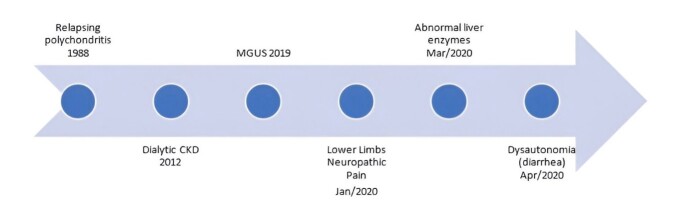
Photomicrograph of the bone marrow. **A –** Congo red staining producing green birefringence under polarized light (200X); **B –** presence of amyloid material among the hematopoietic cells (H&E, 200X).

She was in outpatient follow-up due to relapsing polychondritis for the past 30 years, with nasal and auricular chondritis and sensorineural hearing loss, without immunosuppression for 15 years. She also was undergoing regular peritoneal dialysis due to multifactorial end-stage chronic kidney disease. Additionally, she had been diagnosed with low-risk monoclonal gammopathy of undetermined significance (MGUS) IgG lambda, diagnosed 2 years prior, with a 0.4 g/dL monoclonal peak on the last serum protein electrophoresis, without abnormal free light chain ratio or clinical relevance suggestive of multiple myeloma (Figure 2).

On physical examination, she showed no signs of dehydration. Neurological examination revealed lower limbs symmetrical and discrete weakness (muscle strength grade 4), abolished ankle jerk reflex, hypotonic and symmetric peripheral reflexes, associated with plantar superficial hypoesthesia and bilateral lateral allodynia, suggesting fibular nerve involvement. The syndromic neurological diagnosis of painful small and large fiber crural peripheral neuropathy was made.

Liver and canalicular tests remained steady without great alterations during the stay.

Electroneuromyography confirmed length-dependent symmetrical sensorimotor polyneuropathy with axonal predominance. Complementary tests showed a stable monoclonal peak with a kappa/lambda free light chain ratio of 0.34. Echocardiography revealed an interventricular septum thickness of 1.4 cm (reference value 0.7 – 1.1 cm) and a left ventricular mass index of 83 to 112 g/m^2^ (reference value < 96 g/m^2^). Additionally, laboratory results showed a troponin T level of 0.512 mcg/L (reference value < 0.014 mcg/L) and an NT-proBNP level of 146,000 ng/L (reference value < 125 ng/L).

Histologic evaluation of abdominal fat pad aspirate and bone marrow biopsy were negative for amyloid deposition. Due to high suspicion, a second bone marrow biopsy was warranted, with a positive result for amyloid protein (Figures 1A and 1B).

**Figure 3:**
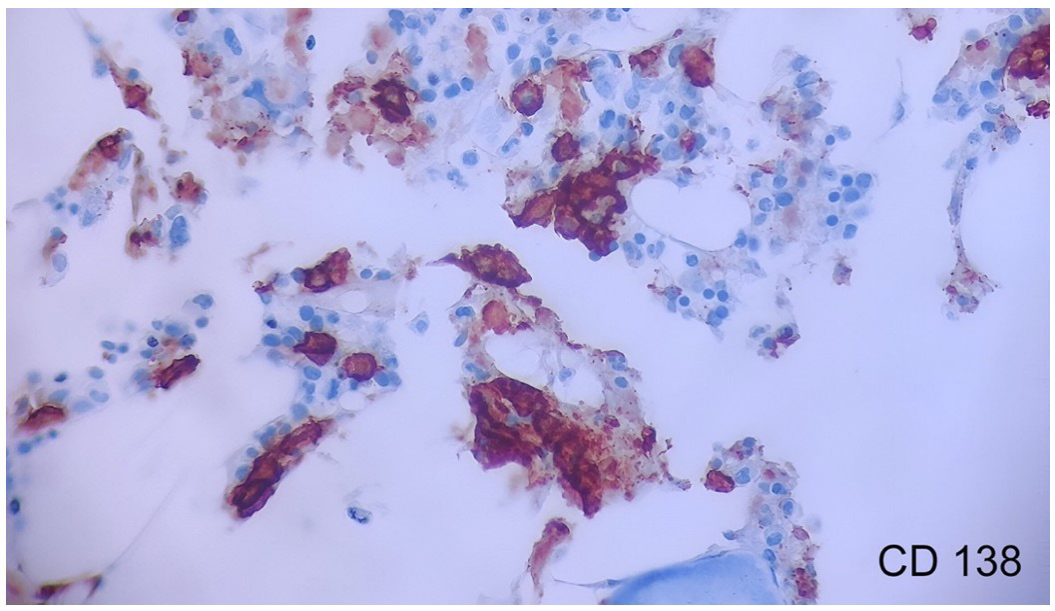
Photomicrograph of the bone marrow showing plasma cells positive for CD138 (400X).

The further evaluation found that 6% of CD138 positive plasma cells with a predominance of lambda light chains (Figure 3).

We approached our patient’s polyneuropathy with standard therapy for neuropathic pain, consisting of systemic amitriptyline and gabapentin combined with an ointment of lidocaine + amitriptyline as a topic agent with good response.

The generalized progressive autonomic failure was managed with pharmacological agents that increase intravascular volume, in this case, fludrocortisone. This synthetic mineralocorticoid that increases renal sodium reabsorption and expands plasma volume was prescribed initially, progressing to increasing doses of vasopressors such as norepinephrine and vasopressin during the patient's ICU stay.

The patient presented unfavorable evolution, with progressive dysautonomia, being admitted to the ICU, culminating in refractory circulatory shock, and undergoing an empirical broad spectrum of antibiotic therapy. After a few days, she presented pulseless ventricular tachycardia, culminating in her death. The patient was referred for an autopsy with family consent for diagnostic confirmation and educational purposes. However, due to the COVID-19 pandemic, our service could only perform autopsies on COVID-positive patients, and thus, the procedure could not be carried out.

The type of amyloid protein was subsequently identified through laser microdissection of amyloid fibrils followed by liquid chromatography–tandem mass spectrometry as AL-lambda (amyloid light-chain) amyloidosis.

## DISCUSSION

There are more than 30 subtypes of amyloidosis with a similar clinical picture and different treatment regimens and prognosis. Because our patient had different comorbidities that could lead to divergent types of amyloidosis with symptoms that overlap, confirming the presence and type of amyloid fibril responsible for the condition was mandatory.

Primary amyloidosis is related to low-grade plasmacyte dyscrasias, which can secrete, among other proteins, light-chain immunoglobulins. The clinical presentation includes renal dysfunction, proteinuria, preserved ejection fraction heart failure, hepatosplenomegaly with intrahepatic cholestasis, carpal tunnel syndrome, cardiovascular, and gastrointestinal dysautonomia. It is difficult to diagnose in the early stages of the disease and requires high suspicion for the correct diagnosis and management. Furthermore, our patient presented a stable monoclonal peak with a kappa/lambda free light chain ratio (KLR) of 0.34, which might have been overlooked due to its magnitude. Nevertheless, as light chains are renally excreted, patients with chronic kidney disease have impaired light chain clearance, with worsening in the normally more efficient kappa (compared with lambda) light chain clearance, and, because kappa light chains are also typically produced at a higher rate than lambda. Modestly elevated KLRs in patients with chronic kidney disease may be due to the perturbed balance of clearance and production.^12^ Our patient, despite being dialytic, had a low KLR, suggesting that, even though she had a discrete ratio alteration, it should be worth noting.

AA amyloidosis is characterized by depositing amyloid fibrils composed of serum amyloid A protein (SAA). It typically occurs due to chronic inflammatory conditions, such as rheumatoid arthritis, chronic infections, or inflammatory bowel disease. In this case, the patient's history of relapsing polychondritis could have contributed to the development of AA amyloidosis.^13^

The patient's chronic kidney disease requiring peritoneal dialysis for eight years raises the possibility of Aß2M amyloidosis, which is associated with the deposition of amyloid fibrils composed of ß2-microglobulin protein and occurs primarily in patients on long-term dialysis who have end-stage renal disease.^10^

The current basis for the treatment of amyloidosis is the so-called precursor-product concept, associated with standard clinical support regarding the disease’s manifestations. The central idea of this concept is that further growth of amyloid deposits will stop when the supply of necessary precursors is stopped. Thus, it is important to diagnose amyloidosis early and start treatment as early as possible to stabilize the disease and prevent ongoing progression.^2^

## CONCLUSION

Amyloidosis encompasses a heterogeneous group of over 30 diseases characterized by the deposition of misfolded proteins in various organs, posing challenges in diagnosis and treatment. This article highlights the crucial role of precise identification in guiding appropriate therapeutic strategies. The presented case of a patient with multiple comorbidities exemplifies the intricate diagnostic process, underscoring the need to discern between various amyloidosis subtypes. Despite therapeutic interventions, the unfortunate outcome highlights the challenges of managing advanced stages of amyloidosis and underscores the critical need for early detection to address these complex conditions effectively. The article advocates for ongoing research efforts to enhance our understanding and refine therapeutic approaches for these complex diseases.

### Learning points

- Importance of considering multiple types of amyloidosis: This case report emphasizes the significance of considering various types of amyloidosis, including AA amyloidosis, AL amyloidosis, and Aß2M amyloidosis, in the differential diagnosis of patients with clinical features suggestive of amyloidosis. The complexity of amyloidosis warrants a comprehensive diagnostic approach, including repeated biopsies and specialized testing, to accurately identify the specific amyloid protein involved.
- Diagnostic Challenges and the Role of Repeat Biopsies: The case highlights the diagnostic challenges associated with amyloidosis, particularly in cases with initial negative histologic evaluations. It underscores the importance of maintaining a high index of suspicion and the potential need for repeat biopsies when clinical suspicion remains strong.
- Multidisciplinary Approach and Timely Management: Primary amyloidosis with multi-organ involvement requires a multidisciplinary approach involving various specialties, including hematology, nephrology, neurology, and cardiology. Early recognition, accurate diagnosis, and prompt initiation of appropriate management strategies, such as chemotherapy, stem cell transplantation, and supportive care, are crucial for optimizing patient outcomes. This case underscores the importance of timely and coordinated care to address the multi-faceted manifestations and complications of primary amyloidosis.
